# Editorial for special issue—human precision cut lung slices: an Ex vivo platform for therapeutic target discovery and drug testing in lung disease

**DOI:** 10.1186/s12931-024-02756-8

**Published:** 2024-03-23

**Authors:** Cynthia J. Koziol-White, Reynold A. Panettieri Jr.

**Affiliations:** Rutgers Institute for Translational Medicine and Science, Rutgers University, 89 French Street, Room 4268, New Brunswick, NJ 08901 USA

**Keywords:** Precision cut lung slices, Respiratory therapeutics, Airway physiology in precision cut lung slices

## Abstract

Publications utilizing precision cut lung slices (PCLS) steadily increased from the 1970’s, with a significant increase in 2010, to tripling by 2023. PCLS have been used to study a vast array of pulmonary diseases and exposures to pathogens and toxicants to understand pathogenesis of disease but also to examine basic cellular mechanisms that underly lung biology. This Special Issue will highlight new, exciting, and novel research using PCLS, while acknowledging the substantial fund of knowledge that has been gained using this platform.

We are excited to offer a Special Issue for Respiratory Research titled “Human Precision Cut Lung Slices: An Ex Vivo Platform for Therapeutic Target Discovery and Drug Testing in Lung Disease.” Since the 1970s, PCLS have served as invaluable tools in the study of various facets of lung disease, spanning toxicology, metabolism (both drug and cellular), drug discovery, lung cancer, obstructive and fibrotic lung diseases, respiratory pathogen exposure, breathing mechanics, and vascular lung diseases.

PCLS represents a pioneering, multi-dimensional platform that enables researchers to delve into the intricacies of lung biology and physiology. From unraveling the fundamental mechanisms underlying breathing mechanics to deciphering the immunological aspects of lung tissue and unraveling the pathogenesis of lung diseases, PCLS offers a comprehensive view.

Researchers have leveraged lungs from diverse species to deepen our understanding of lung biology. PCLS stands as a pivotal platform for therapeutic drug discovery and testing aimed at reversing or preventing the pathogenesis of various lung diseases.

This Special Issue will feature a diverse array of articles, including short reports, full-length research articles, and comprehensive reviews of current literature, all focusing on the pivotal role of PCLS in unraveling both fundamental and pathological mechanisms in lung biology. We enthusiastically welcome groundbreaking studies that push the boundaries of PCLS utilization in the exploration of lung biology and disease.

## Data Availability

No datasets were generated or analysed during the current study.

## List of abbreviations

PCLS: Precision cut lung slices

